# Enhanced Photoluminescent Properties and Crystalline Morphology of LiBaPO_4_:Tm^3+^ Phosphor through Microwave Sintering Method

**DOI:** 10.3390/ma9050356

**Published:** 2016-05-12

**Authors:** Hsuan-Lin Lai, Min-Hang Weng, Ru-Yuan Yang, Shoou-Jinn Chang

**Affiliations:** 1 Institute of Microelectronics, Department of Electrical Engineering, Advanced Optoelectronic Technology Center, National Cheng Kung University, Tainan 701, Taiwan; shiuannlin@hotmail.com (H.-L.L.); changsj@mail.ncku.edu.tw (S.-J.C.); 2 Medical Devices and Opto-Electronics Equipment Department, Metal Industries Research and Development Center, Kaohsiung County 811, Taiwan; mhweng@mail.mirdc.org.tw; 3 Graduate Institute of Materials Engineering, National Pingtung University of Science and Technology, Pingtung County 912, Taiwan

**Keywords:** phosphors, LiBaPO_4_, microwave, luminescence

## Abstract

An investigation of the photoluminescent properties and crystalline morphology of blue emitting LiBa_1−*x*_PO4:*x*Tm^3+^ phosphors with various concentrations (*x* = 0.005–0.030) of Tm^3+^ ions were synthesized by microwave sintering. For comparison, the LiBa_1−*x*_PO4:*x*Tm^3+^ powders sintered at the same sintering condition but in a conventional furnace were also investigated. LiBaPO_4_ without second phase was formed no matter which furnace was used. More uniform grain size distributions are obtained by microwave sintering. When the concentration of Tm^3+^ ion was *x* = 0.015, the luminescence intensity reached a maximum value, and then decreased with the increases of the Tm^3+^ concentration due to concentration quenching effect. The microwave sintering significantly enhanced the emission intensity of LiBa_1−*x*_PO4:*x*Tm^3+^ phosphors. Additionally, the d-d interaction is the key mechanism of concentration quenching for LiBaPO_4_:Tm^3+^. The chromaticity (*x*, *y*) for all LiBa_1−*x*_PO_4_:*x*Tm^3+^ phosphors are located at (0.16, 0.05), which will be classified as a blue region.

## 1. Introduction

Several advantages of white light-emitting diodes (WLEDs) such as high luminous efficiency, energy-saving, maintenance as well as environmental protection, lead the WLEDs to be called the next-generation solid-state light and to replace traditional incandescent and fluorescent lamps. The combination of blue LED with yellow luminescence from Y_3_Al_5_O_12_:Ce^3+^ is the present strategy to make white light (YAG:Ce^3+^) phosphor materials [1]. Another method to make white light is to combine ultraviolet LED and different colors of phosphors obtained from excitation by ultraviolet LED. Therefore, many red, green and blue phosphors that can be excited by UV light should be developed [2]. Also, phosphors with high luminescent efficiency, stability and low cost are in demand for application in WLEDs.

Phosphates with a general formula like ABPO_4_, where A is a mono-valent cation and B is a divalent cation, are interesting because of their optical, ferroelectric properties, excellent thermal and hydrolytic stability [2,3,4,5,6,7]. Therefore, many studies have concentrated on ABPO_4_ phosphate by solid-state reaction for WLEDs [2,3,4,5,6,7]. Among them, Tm^3+^-doped materials have been widely adopted as blue emitting phosphors because of their intense ^1^D_2_ → ^3^F_4_ emission. The investigation from Li *et al.* has shown that Tm^3+^-doped NaCaPO_4_ phosphor exhibits relatively strong absorption in near-UV region of 356 nm and intense blue emission of 451 nm with excellent color purity [7].

Regarding the sintering process, it has been reported that, when the phosphors were sintered using the microwave energy as the heating sources, the energy can be absorbed immediately and uniformly compared to results from a conventional solid state sintering process [8]. This technique has been applied recently to prepare various oxide phosphors, such as YInGe_2_O_7_:Eu^3+^ [9], BaY_2_ZnO_5_:Eu^3+^ [10], and Sr_2_SiO_4_:Eu^3+^ [11] and LiBaPO_4_:Dy^3+^ [12].

However, to our knowledge, Tm^3+^ ions doped LiBaPO_4_ phosphors prepared by microwave-assisted sintering or conventional sintering have not yet been reported. In addition, the luminescent characteristics of Tm^3+^ ions doped LiBaPO_4_ and the mechanism of energy transfer of Tm^3+^-doped LiBaPO_4_ phosphor has been little-reported until now. Therefore, in this paper, the luminescent characteristics, microstructure properties and mechanism of energy transfer of LiBaPO_4_:Tm^3+^ phosphors prepared using different sintering processes are investigated.

## 2. Results and Discussion

### 2.1. Structure

**Figure 1:**
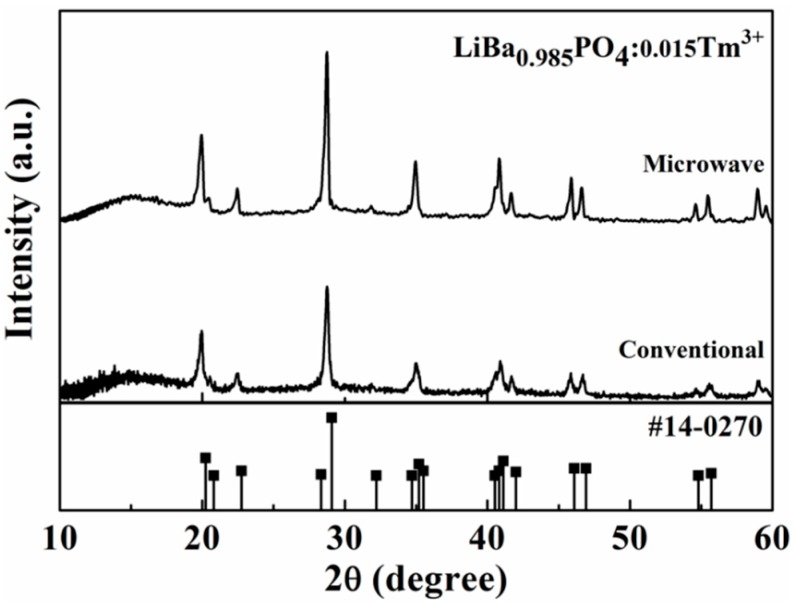
X-ray diffraction patterns X-ray diffraction patterns of LiBa_0.985_PO_4_:0.015Tm^3+^ compared by sintered at 1200 °C for 3 h in different furnace.

Figure 1 shows the X-ray diffraction patterns of LiBa_0.985_PO_4_:0.015Tm^3+^ sintered at 1200 °C for 3 h in different furnace, respectively. According to the Joint Committee on Powder Diffraction Standards (JCPDS #14-0270), the LiBaPO_4_ has a hexagonal crystal structure with a space group P6_3_ [12], and all patterns of LiBa_0.985_PO_4_:0.015Tm^3+^ phosphors with different sintering furnace can be indexed and match the reference [13] and no other second phase or starting material is observed. The XRD result implied that the full-width at half-maximum (FWHM) of LiBa_0.985_PO_4_:0.015Tm^3+^ phosphors prepared using microwave sintering is smaller than that prepared using the conventional one, indicating that the crystallinity of LiBa_0.985_PO_4_:0.015Tm^3+^ was improved by microwave sintering. It is known that a long sintering time causes grain growth, resulting in good crystallinity. However, at the same sintering condition (1200 °C, 3 h) with a different sintering method, the heat through conventional method is indirect, but in a microwave furnace, the material is rapidly heated both internally and externally. The heat generated within the material, and the susceptors provided the heat to the specimen externally by thermal conduction [9]. High sintering efficiency could be obtained through microwave sintering, resulting in good crystallinity.

**Figure 2:**
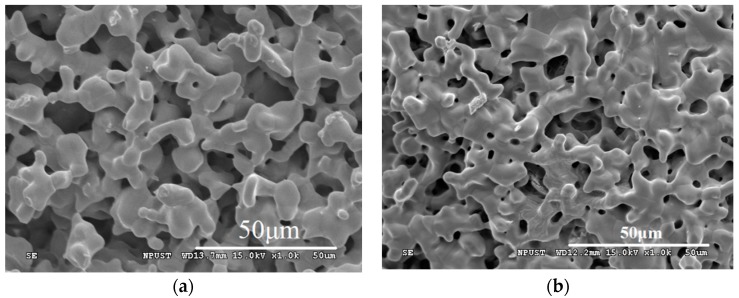
(**a**) SEM image of LiBa_0.985_PO_4_:0.015Tm^3+^ phosphor sintered at 1200 °C for 3 h in microwave furnace; (**b**) SEM image of LiBa_0.985_PO_4_:0.015Tm^3+^ phosphor sintered at 1200 °C for 3 h in conventional furnace.

Figure 2a,b shows typical SEM micrographs for LiBa_0.985_PO_4_:0.015Tm^3+^ sintered in microwave furnace and in conventional furnace at 1200 °C for 3 h, respectively. The microstructures of the LiBa_0.985_PO_4_:0.015Tm^3+^ powders changed significantly through different sintering processes. The shapes of the particles are not very different from one another, but the grain size distribution from microwave sintered powder is uniform. The grain distribution of conventionally sintered powder reveals the agglomeration of particles. Additionally, the particle sizes of LiBa_0.985_PO_4_:0.015Tm^3+^ phosphors are in the range of 9–11 μm.

### 2.2. Photoluminescence Properties

**Figure 3:**
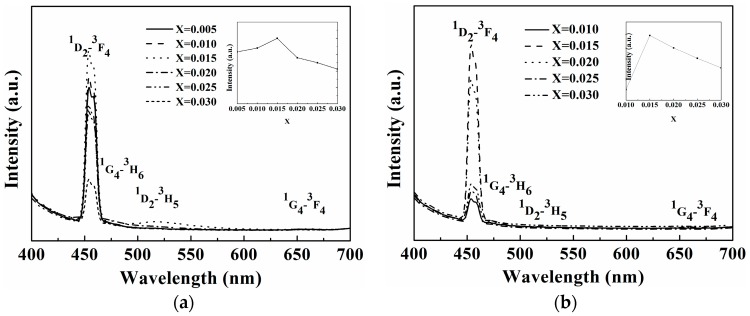
(**a**) Emission spectra of LiBa_1−*x*_PO_4_:*x*Tm^3+^ phosphors with different concentrations of Tm^3+^ ions sintered at 1200 °C for 3 h in microwave furnace (λ*_ex_* = 359 nm). The inset on the top right is the tendency of the blue emission intensity (454 nm) for LiBa_1−*x*_PO4:*x*Tm^3+^ at 0.005 < x ≤ 0.030; (**b**) Emission spectra of LiBa_1−*x*_PO_4_:*x*Tm^3+^ phosphors with different concentrations of Tm^3+^ ions sintered at 1200 °C for 3 h in convemtional furnace (λ*_ex_* = 359 nm). The inset on the top right is the tendency of the blue emission intensity (454 nm) for LiBa_1−*x*_PO4:*x*Tm^3+^ at 0.010 < x ≤ 0.030.

Figure 3a,b shows the emission spectra of LiBa_0.985_PO_4_:0.015Tm^3+^ phosphors with different concentrations of Tm^3+^ ions using different sintering process, respectively. LiBa_0.985_PO_4_:0.015Tm^3+^ phosphors can be effectively excited by NUV LED because all the LiBa_0.985_PO_4_:0.015Tm^3+^ phosphors are excited by a xenon lamp at a wavelength of 359 nm. The inset of Figure 3a shows the tendency of the blue emission intensity (454 nm) for LiBa_1−*x*_PO4:*x*Tm^3+^ at 0.005 < *x* ≤ 0.030 sintered in a microwave furnace. The inset of Figure 3b shows the tendency of the blue emission intensity (454 nm) for LiBa_1−*x*_PO4:*x*Tm^3+^ at 0.010 < x ≤ 0.030 sintered in conventional furnace. Both of the emission spectra of the LiBa_1−*x*_PO_4_:*x*Tm^3+^ phosphors display four emission bands at 454, 476, 513, and 660 nm, respectively corresponding to the ^1^D_2_ → ^3^F_4_, ^1^G_4_ → ^3^H_6_, ^1^D_2_ → ^3^H_5_ and ^1^G_4_ → ^3^F_4_ transitions of Tm^3+^ ions.

**Figure 4:**
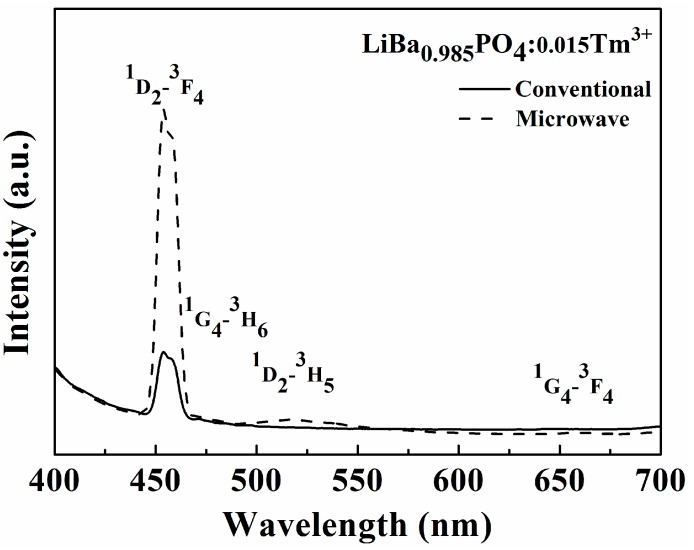
The emission spectra of LiBa_0.985_PO4:0.015Tm^3+^ phosphors with different sintering process.

Figure 4 shows the emission spectra of LiBa_0.985_PO_4_:0.015Tm^3+^ phosphors with different sintering processes. It is shown that LiBa_0.985_PO_4_:0.015Tm^3+^ phosphor prepared by microwave sintering has much higher luminescent intensity than that of a conventionally sintered one. Referring to Figure 1, the crystallinity of LiBa_0.985_PO_4_:0.015Tm^3+^ phosphors prepared by microwave sintering is better than that using conventionally sintering so that higher luminous intensity is expected. As the concentration of Tm^3+^ ion increased, the probability of the energy transfer among Tm^3+^ ions also increased. The luminescence intensity reached a maximum when the concentration of Tm^3+^ ion was at 0.015, and then decreased with the increases of the Tm^3+^ concentration due to the concentration quenching effect.

Blasse proposed the critical transfer distance (*Rc*) to realize the mechanism of energy transfer in phosphors whereby *Rc* is about equal to twice the radius of a sphere with the volume as shown in Equation (1) [14,15,16].

(1) $$Rc=2{\left[\frac{3V}{4\pi x_{c}N}\right]}^{1/3}$$

**Figure 5:**
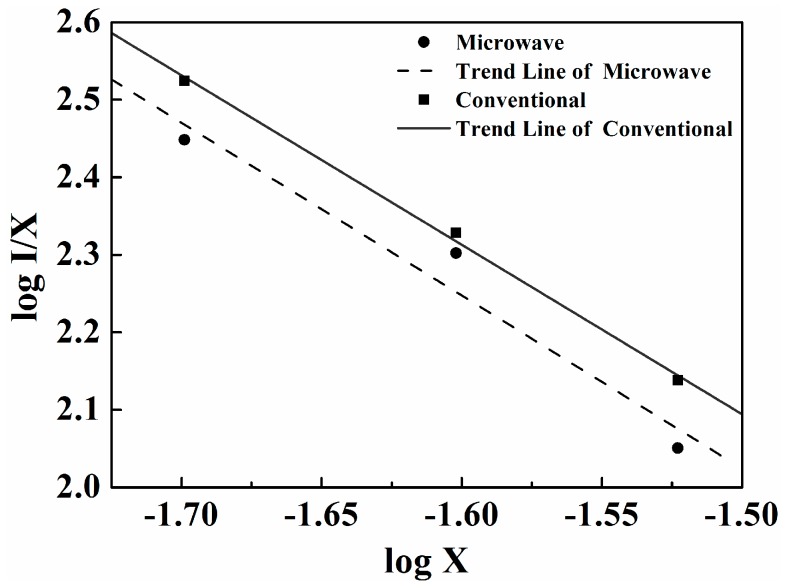
The curve of log (*I*/*x*) *vs.* log (*x*) in LiBa_1−*x*_PO_4_:*x*Tm^3+^ phosphors with doped Tm^3+^ concentration at *x* = 0.02, 0.025, 0.03 using different sintering process.

In which *x_c_* is the critical concentration, *N* is the number of cations in the unit cell and *V* is the volume of the unit cell. The unit cell volume *V* of LiBaPO_4_ is 0.391577 nm^3^ and the critical concentration *x_c_* is 0.015, resulting from the maximum intensity of LiBa_1−*x*_PO_4_:*x*Tm^3+^ at *x* = 0.015. The number of host cations in the unit cell of LiBaPO_4_ is 4. Based on the above values, the critical distance of energy transfer *Rc* is calculated as 2.3186 nm. Exchange interaction, radiation reabsorption, or multipole-multipole interaction and so-called non-radiative energy transfer between different Tm^3+^ ions may have happened. When the typical critical distance is approximately 5 Å, the exchange interaction is generally responsible for the energy transfer of forbidden transitions [16]. When the sensitizer and activator coexist in phosphor system, the mechanism of radiation reabsorption occurs due to broad overlap between excitation and emission spectra. In this study, there is no overlap between excitation and emission spectra of LiBa_1−*x*_PO_4_:*x*Tm^3+^ phosphor. Besides, the critical distance *Rc* (2.3186 nm) of LiBa_1−*x*_PO_4_:*x*Tm^3+^ phosphor is larger than 5 Å. Therefore, the energy transfer mechanism between Tm^3+^ ions in LiBaPO_4_ phosphor could be suggested by multipole-multipole interaction from Dexter's theory [16]. If the energy transfer takes place between the same sorts of Tm^3+^ ions, the multipole-multipole interaction effect can be determined from of the difference of the emission intensity according to the emitting level with multipolar interaction. The emission intensity (*I*) per Tm^3+^ ion can be calculated by the Equation (2) [14,15,16]:

(2) $$I/x=K{\left[1+\beta {\left(x\right)}^{Q/3}\right]}^{-1}$$

where *x* is the activator concentration; *Q* = 6, 8 or 10 for dipole–dipole (*d*-*d*), dipole-quadrupole (*d*-*q*) or quadrupole-quadrupole (*q*-*q*) interaction, respectively; and *K* and β are constants for a given host crystal with the same excitation condition. The doped Tm^3+^ concentration, which is not less than the critical concentration (*i.e*., *x* = 0.02, 0.025, 0.03), is used to determine the dependence of the emission intensity of LiBaPO_4_:Tm^3+^ phosphor excited at 359 nm. As shown in Figure 5, the dependence of log (*I*/*x*) on log (*x*) in microwave sintering is linear and the slope is −2.231. The value of *Q* can be calculated as 6.693. In addition, the dependence of log (*I*/*x*) on log (*x*) in conventional sintering is linear and the slope is −2.186. The value of *Q* can be calculated as 6.558. Both are approximately equal to 6 based on Equation (2). Therefore, the *d*-*d* interaction is the major mechanism for concentration quenching of the LiBaPO_4_:Tm^3+^ phosphor no matter what sintering method was used.

**Figure 6:**
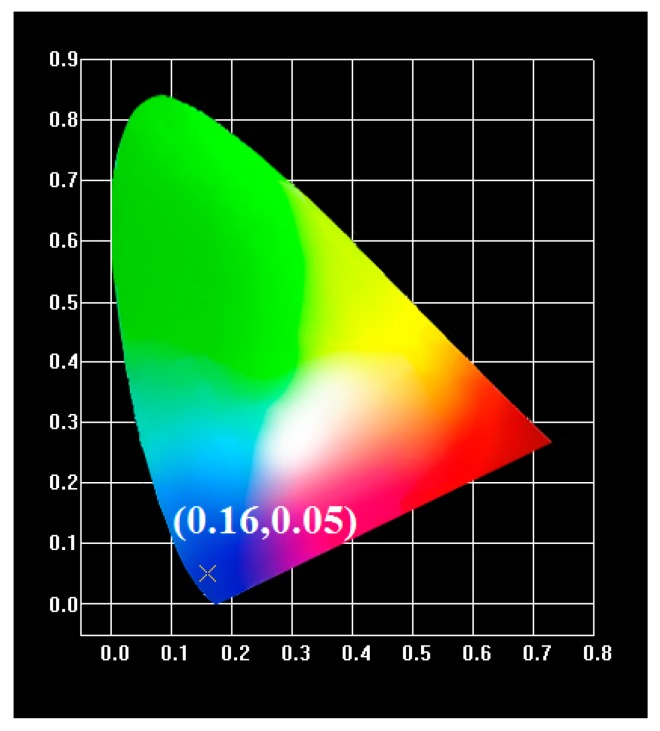
The CIE1931 chromaticity diagram of LiBa_0.985_PO4:0.015Tm^3+^ phosphors using different sintering process.

It is known that color coordinates could be established by Commission International de l’Eclairage (CIE) 1931 according to a two-dimensional graphical representation of any color perceptible by the human eye. The CIE coordinates could be obtained through commercial software by converting the data of photoluminescence emission spectrum. Figure 6 shows all LiBa_0.985_PO_4_:0.015Tm^3+^ phosphors having the same chromaticity (*x*, *y*) coordinates located in the blue region (0.16, 0.05). Therefore, if we choose appropriate green and red phosphors mixed with the LiBaPO_4_:Tm^3+^ onto the ultraviolet LED chip, the goal to form WLEDs could be achieved.

## 3. Experimental Procedure

### 3.1. Sample Preparation

The LiBa_1−*x*_PO_4_:*x*Tm^3+^ phosphors used Li_2_CO_3_ (99.94%), BaCO_3_ (99.9%), NH_4_H_2_PO_4_ (98%), and Tm_2_O_3_ (99.9%) powders as starting materials and different concentrations of Tm^3+^ ions (*x* = 0.005–0.03) were chosen as a variable parameter. The starting materials were mixed using alcohol as a solvent and then ball-milled for 1 h with zirconia balls. After drying, the mixed powders were sintered in a microwave furnace and conventional furnace to form LiBa_1−*x*_PO_4_:*x*Tm^3+^ phosphors. As in the case of microwave sintering, a microwave furnace (Therm Wave Mod. III) with a continuously variable power of 2.45 GHz microwaves up to 1.3 kW was used. Silicon carbide (SiC), having a very strong heating response to 2.45 GHz microwaves, was used as a susceptor to provide indirect heating of the powders. For comparison, the sample was also sintered at 1200 °C for 3 h in conventional furnace under an air atmosphere with the heating rate controlled at 10 °C/min. The average heating rate of microwave furnace was greater than 100 °C/min. After sintering, the phosphor samples were cooled to room temperature and then characterized.

### 3.2. Characterization

The crystalline phases of the phosphors were identified by X-ray diffraction (XRD, model D8 Advance, Bruker Axs Gmbh, Karlsruhe, Germany) with CuKα radiation of λ = 1.54 Å using a Ni filter, and a secondary graphite monochromator. A scanning range of 2θ = 10°~60° was used with a step of 0.03° and 0.4 s as a per-step count time. The particle morphology of phosphors was identified by scanning electron microscopy (SEM; model S-3000N, Hitachi, Ltd., Tokyo, Japan). Additionally, the excitation, emission spectra and the color coordinates and the Commission International de l’Eclairage (CIE) information were obtained using a photoluminescence spectrophotometer (PL, model FP-6000, JASCO Corporation, Tokyo, Japan) with a 150 W xenon lamp as the light source.

## 4. Conclusions

In this paper, LiBa_0.985_PO_4_:0.015Tm^3+^ phosphors with various sintering process were successively synthesized at 1200 °C for 3 h. XRD results indicate that pure LiBa_0.985_PO_4_:0.015Tm^3+^ phosphor phase was formed. The major emission peak centered at 454 nm corresponds to the ^1^D_2_ → ^3^F_4_ transition and the maximum photoluminescence intensity appeared at the Tm^3+^ concentration of 0.015. Under the microwave sintering, good crystallinity and uniform grain size distributions are obtained because microwave energy provides the material rapidly heated both internally and externally. The *d*-*d* interaction plays a major role in the mechanism of concentration quenching of LiBaPO_4_:Tm^3+^ phosphor based on the theoretical calculation no matter what sintering method was used. Additionally, WLEDs could be achieved by mixing appropriate green and red phosphors with the LiBaPO_4_:Tm^3+^ onto the ultraviolet LED chip.
